# Editorial: Molecular regulation of seed development and storage reserve metabolism in crops

**DOI:** 10.3389/fpls.2023.1348252

**Published:** 2024-01-09

**Authors:** Sehrish Manan, Saqib Bilal

**Affiliations:** ^1^ Biofuels Institute, School of the Environment and Safety Engineering, Jiangsu University, Zhenjiang, China; ^2^ Natural and Medical Sciences Research Center, University of Nizwa, Nizwa, Oman

**Keywords:** embryogenesis, seed development, storage reserve metabolism, stress regulation, transcription factor

The life cycle of plants consists of distinct growth phases that are regulated by genetic programs. One important stage is the transition from vegetative growth to reproduction, which involves the regulation of several genes (Raihan et al., 2021). Another crucial phase is seed development and maturation, which is essential for the reproductive success of plants (Eskandari, 2012). During this phase, seeds accumulate storage reserves such as proteins, triacylglycerols (TAGs), and starch, which impact seed quality and viability (Manan et al., 2017). During seed maturation, desiccation and dormancy induction are also important physiological phenomena. Understanding the genetic mechanisms behind seed development and storage substances is important for agricultural production as it influences crop yield and nutritional value.

The global challenge of feeding a growing population requires the cultivation of high-yield crops. To achieve this, researchers are conducting extensive studies to better understand plant development (Hilty et al., 2021), nutritional quality enhancement (Gaikwad et al., 2020), and the potential for increased yield (Edgerton, 2009). The goal is to develop crop varieties that not only have higher yields but also greater nutritional value. To achieve this, further exploration of plant development, embryogenesis, and the mobilization of storage substances in important crops such as wheat, soybean, maize, and rice, is still required (Guo et al., 2022). Additionally, there are several unanswered questions regarding the relationship between protein, oil, and carbohydrate biosynthesis in seeds (Manan et al., 2023). Additionally, the coordination of transcription factors and their downstream target genes and how they regulate various phytohormones such as abscisic acid, gibberellin, cytokinin etc. during plant development and reproduction (Brocard-Gifford et al., 2003; Manan and Zhao, 2021).

This Research Topic in Frontiers in Plant Science, Sections ‘Plant Metabolism and Chemodiversity’ and ‘Crop and Product Physiology’ discusses the genetic basis of seed growth, maturation, and dormancy. This Research Topic features 7 articles, including five original research articles and two reviews contributed by experts in the field. These articles mainly focus on seed biopriming, seed development, molecular regulation of seed storage components, and impact of stress on plant yield.

Over the past 10,000 years, the domestication and selective breeding of seed plants have played a crucial role in sustaining the human population. However, our understanding of the biological mechanisms that regulate seed development, evolution, physiology, and yield is still incomplete (Gupta et al., 2022). During seed maturation, various changes occur in the seed tissues, resulting in the production of storage compounds. Biosynthesis, accumulation of storage reserves, plant development, plant growth and regulation of physiological characteristics of plant such as height, leaf shape etc. are all interlinked processes. Cardenas-Conejo et al. found enriched pathways related to triterpenes, sesquiterpenes, and cuticular wax biosynthesis in three *B. orellana* accessions, indicating a complex and highly coordinated process of metabolite biosynthesis. Advancements in sequencing technologies, as demonstrated by Tayade et al., have revolutionized functional genomic research, providing insights into the genetic and molecular basis of yield traits in economically important crops. Furthermore, technological advancements and advanced analytical instrumentation have greatly enhanced our understanding of complex biological processes. Metabolites play a crucial role in regulating physiological mechanisms and understanding of biological phenomena. Integration of metabolomics with sequencing data, as discussed by Oh et al., can provide more substantial evidence how genetic variants respond to a specific stress and its effect on the metabolic processes. The application of metabolomics can be instrumental in ensuring the safety and effectiveness of transgenic crops.

Abiotic stresses, such as drought, cold, and heat, directly affect plant development and yield (Fahad et al., 2017). The involvement of phytohormones, such as auxin (Wang and Irving, 2011), gibberellins (Vishal and Kumar, 2018), cytokinins (Wu et al., 2021), and abscisic acid (Emenecker and Strader, 2020), in regulating seed size and yield is well-established. Phytohormones act as chemical messengers, helping plants withstand both abiotic and biotic stresses by regulating a complex system (Zhu, 2016). The biopriming technique aids in selective absorption of soil nutrients and maintains membrane stability and chlorophyll synthesis (Chakraborti et al., 2022). Shaffique et al. demonstrated that inoculating the SH-8 bacteria in the rhizosphere is a sustainable strategy for improving drought tolerance through growth promotion, phytohormone and antioxidant production. This strategy increases plant biomass and germination while minimizing crop oxidative stress. Excessive UV radiation can detrimentally impact plant growth and metabolism. Studies have shown that even low levels of UV-B exposure can trigger gene activity and affect agronomic and physiological parameters in plants, including plant height, photosynthetic rate, and nutrient synthesis (O’Hara et al., 2019; Rodríguez-Calzada et al., 2019). Additionally, it increases the expression of structural genes for anthocyanin synthesis, leading to higher nutrient content in tubers (Henry-Kirk et al., 2018). Wu et al. found that appropriate UV-B radiation enhanced oxidative stress tolerance and improved the yield and quality of potato tubers.

Nickel (Ni) is a micronutrient necessary for plant growth because it is active site of urease enzyme; however, its high concentrations can be toxic (Amjad et al., 2020). In plants two forms of urease are present one in seeds (highly active) and other in vegetative tissues (less active) and play important role in nitrogen cycling, synthesis of amino acids and other nitrogen compounds. (Fabiano et al., 2015). A study has shown that elevated Ni levels in plants lead to oxidative damage, resulting in increased hydrogen peroxide and malonaldehyde levels (Baccouch et al., 2001). However, sweet potato plants have been observed to tolerate moderate Ni treatment (up to 15 mg/L) by reducing oxidative stress, as observed by Kumar et al., indicating that low Ni-contaminated soil benefits sweet potato growth. Furthermore, sweet potato can potentially be utilized as a phytoremediator in moderately Ni-contaminated soil. Initial phases of plant growth highly depend on soil properties such as moisture, porosity and nutrient supply. Plant growth-promoting rhizobacteria (PGPR) helps to regulate nitrogen fixation, production of growth hormones like indole acetic acid, production of exopolysaccharides, and lower soil pH to provide a sustainable agroecosystem under normal and stress conditions. Similarly, biochar (BC) has the potential to enhance soil quality, mitigation of organic and inorganic pollutants to alleviate salinity, heavy metals and drought. In maize the addition of corn cob BC increases the seed germination, seedling growth and improves the antioxidant activity of plants (Ali et al., 2021). Gul et al., investigated the effects of BC and PGPR, both individually and in combination, on the growth, physiology, and biochemical traits of barley plants under drought stress. The results demonstrated that the combine use of PGPR and BC significantly alleviated the adverse effects of drought, and led to improvement in shoot length, biomass, seed germination, and physiological traits. The synergistic interaction between PGPR and BC also increased the activity of antioxidant enzymes and enhanced soil fertility. These findings suggest that the implementation of BC and PGPR can enhance crop production in water-deficient areas.

In summary, this Research Topic explores the essential information related to seed metabolism. It also presents innovative approaches such as seed biopriming, BC and PGPR treatment to improve stress tolerance, as well as low UV-B treatment to boost crop yield.

## Author contributions

SM: Conceptualization, Data curation, Writing – original draft. SB: Writing – review & editing.

## References

[B1] Ali L. Xiukang W. Naveed M. Ashraf S. Nadeem S. M. Haider F. U.. (2021). Impact of biochar application on germination behavior and early growth of maize seedlings: insights from a growth room experiment. Appl. Sci. 11, 11666. doi: 10.3390/app112411666

[B2] Amjad M. Raza H. Murtaza B. Abbas G. Imran M. Shahid M.. (2020). Nickel toxicity induced changes in nutrient dynamics and antioxidant profiling in two maize (Zea mays L.) hybrids. Plants 9, 5. doi: 10.3390/plants9010005 PMC7020203 31861411

[B3] Baccouch S. Chaoui A. El Ferjani E. (2001). Nickel toxicity induces oxidative damage in zea mays roots. J. Plant Nutr. 24, 1085–1097. doi: 10.1081/PLN-100103805

[B4] Brocard-Gifford I. M. Lynch T. J. Finkelstein R. R. (2003). Regulatory networks in seeds integrating developmental, abscisic acid, sugar, and light signaling. Plant Physiol. 131, 78–92. doi: 10.1104/pp.011916 12529517 PMC166789

[B5] Chakraborti S. Bera K. Sadhukhan S. Dutta P. (2022). Bio-priming of seeds: Plant stress management and its underlying cellular, biochemical and molecular mechanisms. Plant Stress 3, 100052. doi: 10.1016/j.stress.2021.100052

[B6] Edgerton M. D. (2009). Increasing crop productivity to meet global needs for feed, food, and fuel. Plant Physiol. 149, 7–13. doi: 10.1104/pp.108.130195 19126690 PMC2613695

[B7] Emenecker R. J. Strader L. C. (2020). Auxin-abscisic acid interactions in plant growth and development. Biomolecules 10, 281. doi: 10.3390/biom10020281 32059519 PMC7072425

[B8] Eskandari H. (2012). Seed quality variation of crop plants during seed development and maturation. Int. J. Agron. Plant Production 3, 557–560.

[B9] Fabiano C. Tezotto T. Favarin J. L. Polacco J. Mazzafera P. (2015). Essentiality of nickel in plants: a role in plant stresses. Front. Plant Sci. 6. doi: 10.3389/fpls.2015.00754 PMC4585283 26442067

[B10] Fahad S. Bajwa A. A. Nazir U. Anjum S. A. Farooq A. Zohaib A.. (2017). Crop production under drought and heat stress: plant responses and management options. Front. Plant Sci. 8. doi: 10.3389/fpls.2017.01147 PMC5489704 28706531

[B11] Gaikwad K. B. Rani S. Kumar M. Gupta V. Babu P. H. Bainsla N. K.. (2020). Enhancing the nutritional quality of major food crops through conventional and genomics-assisted breeding. Front. Nutr. 7. doi: 10.3389/fnut.2020.533453 PMC7725794 33324668

[B12] Guo F. Zhang P. Wu Y. Lian G. Yang Z. Liu W.. (2022). Rice LEAFY COTYLEDON1 hinders embryo greening during the seed development. Front. Plant Sci. 13. doi: 10.3389/fpls.2022.887980 PMC9128838 35620685

[B13] Gupta S. Van Staden J. Doležal K. (2022). An understanding of the role of seed physiology for better crop productivity and food security. Plant Growth Regul. 97, 171–173. doi: 10.1007/s10725-022-00827-8

[B14] Henry-Kirk R. A. Plunkett B. Hall M. McGhie T. Allan A. C. Wargent J. J.. (2018). Solar UV light regulates flavonoid metabolism in apple (Malus x domestica). Plant Cell Environ. 41, 675–688. doi: 10.1111/pce.13125 29315644

[B15] Hilty J. Muller B. Pantin F. Leuzinger S. (2021). Plant growth: the what, the how, and the why. New Phytol. 232, 25–41. doi: 10.1111/nph.17610 34245021

[B16] Manan S. Ahmad M. Z. Zhang G. Chen B. Haq B. U. Yang J.. (2017). Soybean LEC2 regulates subsets of genes involved in controlling the biosynthesis and catabolism of seed storage substances and seed development. Front. Plant Sci. 8. doi: 10.3389/fpls.2017.01604 PMC5611487 28979275

[B17] Manan S. Alabbosh K. F. Al-Andal A. Ahmad W. Khan K. A. Zhao J. (2023). Soybean LEAFY COTYLEDON 1: A key target for genetic enhancement of oil biosynthesis. Agronomy 13, 2810. doi: 10.3390/agronomy13112810

[B18] Manan S. Zhao J. (2021). Role of Glycine max ABSCISIC ACID INSENSITIVE 3 (GmABI3) in lipid biosynthesis and stress tolerance in soybean. Funct. Plant Biol. 48, 171–179. doi: 10.1071/FP19260 32877635

[B19] O’Hara A. Headland L. R. Díaz-Ramos L. A. Morales L. O. Strid Å. Jenkins G. I. (2019). Regulation of Arabidopsis gene expression by low fluence rate UV-B independently of UVR8 and stress signaling. Photochem. Photobiol. Sci. 18, 1675–1684. doi: 10.1039/c9pp00151d 31218318

[B20] Raihan T. Geneve R. L. Perry S. E. Rodriguez Lopez C. M. (2021). The regulation of plant vegetative phase transition and rejuvenation: miRNAs, a key regulator. Epigenomes 5, 24. doi: 10.3390/epigenomes5040024 34968248 PMC8715473

[B21] Rodríguez-Calzada T. Qian M. Strid Å. Neugart S. Schreiner M. Torres-Pacheco I.. (2019). Effect of UV-B radiation on morphology, phenolic compound production, gene expression, and subsequent drought stress responses in chili pepper (Capsicum annuum L.). Plant Physiol. Biochem. 134, 94–102. doi: 10.1016/j.plaphy.2018.06.025 29950274

[B22] Vishal B. Kumar P. P. (2018). Regulation of seed germination and abiotic stresses by gibberellins and abscisic acid. Front. Plant Sci. 9. doi: 10.3389/fpls.2018.00838 PMC6019495 29973944

[B23] Wang Y. H. Irving H. R. (2011). Developing a model of plant hormone interactions. Plant Signal Behav. 6, 494–500. doi: 10.4161/psb.6.4.14558 21406974 PMC3142376

[B24] Wu W. Du K. Kang X. Wei H. (2021). The diverse roles of cytokinins in regulating leaf development. Hortic. Res. 8, 1–13. doi: 10.1038/s41438-021-00558-3 34059666 PMC8167137

[B25] Zhu J.-K. (2016). Abiotic stress signaling and responses in plants. Cell 167, 313–324. doi: 10.1016/j.cell.2016.08.029 27716505 PMC5104190

